# Case report: A case of lateral neck mass: ectopic thyroid carcinoma or lymph node metastasis?

**DOI:** 10.3389/fonc.2024.1501297

**Published:** 2025-01-07

**Authors:** Xinran Wei, Ping Yu, Chengcheng Duan, Jingyue Zhong, Xianji Wu, Siqi Xiao, Wenxi Yu, Guang Zhang

**Affiliations:** Department of Thyroid Surgery, Jilin Provincial Key Laboratory of Surgical Translational Medicine, Jilin Provincial Precision Medicine Laboratory of Molecular Biology and Translational Medicine on Differentiated Thyroid Carcinoma, The China-Japan Union Hospital of Jilin University, Changchun, China

**Keywords:** ectopic thyroid gland, ectopic thyroid carcinoma, lateral neck ectopic thyroid, lymph node metastatic cancer, papillary thyroid carcinoma

## Abstract

**Objective:**

To review a rare case of a lateral neck mass pathologically confirmed as an encapsulated papillary thyroid carcinoma in our center for complementing the lack of management of rare cases, and to explore the differentiation of primary ectopic thyroid carcinoma and metastatic disease in the context of the presence of malignant tumors within the situ thyroid gland.

**Methods:**

We searched for studies on lateral neck ectopic thyroid cancer to compare and analyze it with metastatic carcinoma of the thyroid gland in terms of clinical features, imaging manifestations, pathological features at molecular level, and treatment principles.

**Results:**

Based on available data, we concluded that the mass of this patient was consistent with metastatic lateral neck ectopic thyroid carcinoma.

**Conclusion:**

Our findings suggest that differentiating between these two conditions is challenging, as there is currently no reliable methods that can adequately determine their nature. Developing a preoperative diagnostic method would be significant for diagnosing pathologic masses in the lateral neck and selecting appropriate surgical extent and adjuvant therapy.

## 1 Introduction

Ectopic thyroid gland (ETG) is a phenomenon in which thyroid tissue appears outside of its normal anatomical position, most often in the anterior midline of the neck due to an abnormal embryonic descent (1, 2), and when it rarely appears lateral to the carotid sheath, it is referred to as lateral ectopic thyroid (3–5)(Lateral ectopic thyroid).

Lateral ectopic thyroid carcinoma (LETC) has long been a controversial topic and is difficult to distinguish from metastatic well-differentiated thyroid cancer due to the lack of clear molecular mechanisms and specific genes (3–6). Evidence-based guidelines for the optimal treatment of lateral ectopic thyroid carcinoma have not yet been established, and lymph node metastasis is crucial for determining the staging of well-differentiated thyroid carcinoma (WTC) in patients over 55 years of age; therefore (7), an accurate differentiation between the two is important for the selection of surgical scope and the evaluation of adjuvant postoperative treatments.

## 2 Presentation of the case

**Figure 1:**
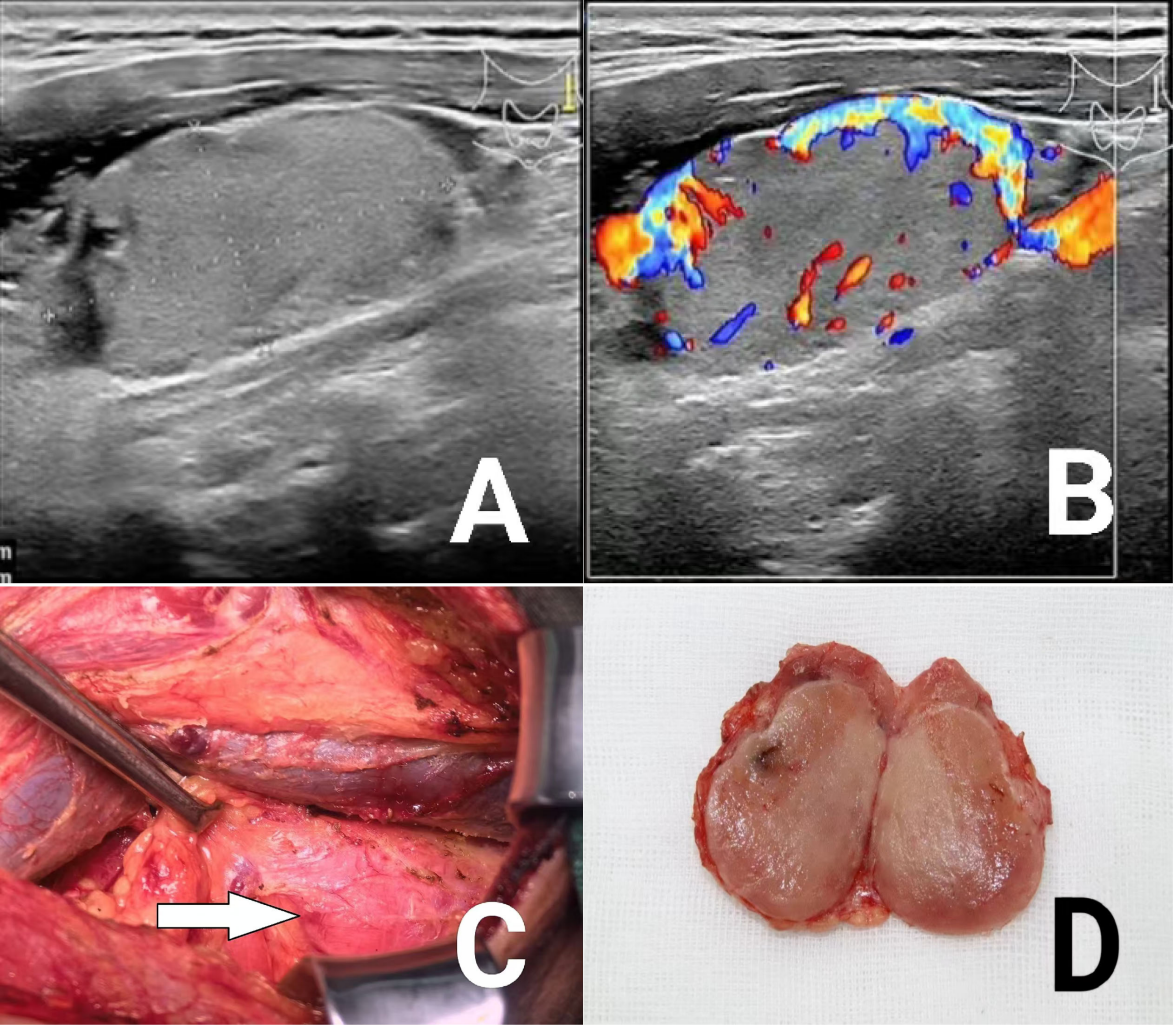
**(A)** Ultrasound image of lateral neck mass; **(B)** Ultrasound blood flow signal image of lateral neck mass; **(C, D)** Intraoperative and gross view of cervical masses.

We report a 48-year-old man who was admitted to our hospital with “a thyroid nodule detected on physical examination for one month”, with no symptoms of hoarseness or dysphagia during the course of the disease. History of malignant tumor and related family genetic history were denied. The clinical examination found a hard, painless, mobile mass measuring about 3.5*2.0 cm was palpable in the left cervical region III. Neck ultrasound: hyperechoic lymph nodes were seen in the left neck region III, about 3.57*1.84 cm in size, with unclear structure, see **Figures 1A, B**. Bilobar thyroid nodule suspicious for malignancy. Fine needle aspiration cytology (FNAc) results of bilobar thyroid nodules: both favored papillary thyroid carcinoma, BRAF V600E (-).Thyroid function was normal:the value of thyrotropin was 1.01mIU/L, free triiodothyronine was 4.96pmol/L, free thyroxine was 17pmol/L, and thyroglobulin was 45.77ng/ml. Combined with the features of mass echogenicity and blood flow signal in the ultrasound image, the mass in region III of the left neck was considered to be possibly (1) metastatic cancer in the lymph nodes; (2) ectopic thyroid.

The patient’s general condition was non-specific, and after signing the informed consent, he underwent enlarged radical thyroidectomy for bilobar carcinoma + left cervical lymph node dissection + resection of the left cervical mass. Intraoperatively, the left neck mass was seen to be similar in appearance to the thyroid gland, with a more richly vascularized surface, but with no connection to the thyroid gland, and after separation and resection, a nodular mass of about 3.5*2.5 cm in size, with a light brown texture on the cut surface and a partially periosteal membrane was seen in the bulk, see **Figures 1C, D**. Rapid intraoperative pathology was sent for examination, suggesting(Left cervical III junction mass)papillary thyroid carcinoma, with a little lymphatic tissue seen at the margins, to be further observed for lymph node metastasis by paraffin section.

Postoperative paraffin pathology findings: (Left cervical III junction mass)encapsulated papillary carcinoma of thyroid (size 3.5*3*2.5cm), lymph node metastatic carcinoma was seen in the margins (1/1), and metastatic foci were 0.05cm in diameter. A total of 36 lymph nodes were cleared from the left cervical sheath and central group, none of which metastasized. Bilobar thyroid nodules were consistent with papillary thyroid carcinoma.

The patient had a good postoperative recovery with grade A healing of the incision and was discharged on medical advice after 3d. After discharge from the hospital, the patient was treated with radioactive iodine as prescribed by the doctor, and now the patient is receiving endocrine suppression therapy with eugenol 100ug/d. There is no recurrence during the current follow-up period of 6 months.

## 3 Discussion

In 1906, Schrager first introduced the concept of “lateral cervical ectopic thyroid” and defined it as “a mass of thyroid tissue with normal or pathological structure at some distance from the normal thyroid gland and without any connection” (3, 5). The concept of thyroid tissue has been recognized in the literature. However, to this day, the origin of lateral cervical ectopic thyroid remains unclear, and the relevant theories mainly include the descent or fusion abnormality of the lateral primordium during embryonic development, the spread of intraoperative tissue from the thyroid gland *in situ*, and the metastasis of highly differentiated thyroid carcinomas (1, 2, 4). With the continuous deepening of the research on papillary thyroid carcinoma, most scholars now associate the origin of ectopic thyroid glands in the lateral neck with metastatic lesions of primary thyroid cancer, and believe that the so-called “ectopic thyroid glands in the lateral neck” basically belong to the metastatic foci of well-differentiated thyroid cancer (3–5, 8, 9), and are mostly located lateral to the carotid sheaths (5). In addition, Shim et al. demonstrated the theory of spontaneous remission of PTC (10, 11), which provides a possible explanation for the metastatic potential of ectopic thyroid carcinoma in the absence of pathological evidence of a primary thyroid tumor. However, in recent years cases of ectopic thyroid gland with benign lateral neck have been reported (9) and in rare cases primary thyroid carcinoma may occur in the ectopic tissue (4). All these controversies make it more difficult to differentiate primary lateral neck ectopic thyroid carcinoma from lymph node metastases.

### 3.1 Clinical characteristics

The clinical features of lateral cervical ectopic thyroid often resemble neck metastases from thyroid malignancies of unknown primary focus (8). Both may have no specific clinical symptoms, and most often present on examination as a palpable, painless, mobile mass in the lateral neck. In this case, the patient came to the clinic due to physical examination without conscious clinical symptoms, and the presence of a thyroid gland *in situ* made it difficult to consider the diagnosis of ectopic thyroid and to differentiate between the two.

### 3.2 Imaging

First, high-resolution ultrasound has become the preferred choice for the initial assessment of ectopic thyroid and its lesions, and for differentiating it from lymph node metastatic carcinoma, by displaying peripheral or internal color flow signals, and by analyzing the imaging characteristics and adjacency of the mass (12): (1) The diversity of ectopic thyroid presentations on ultrasound is related to their coexisting lesions (13). Ectopic tissue without lesions behaves as a characteristic of normal thyroid glands, and ectopic thyroid carcinoma in the lateral neck coexists with the thyroid gland *in situ* in most cases and can present as a solid to cystic lesion in the lateral neck (2, 5), and therefore often results in misdiagnosis. (2) Suspicious signs of metastatic carcinoma in the cervical lymph nodes on ultrasound (14)include clustered hypoechoicity, foci of calcification (predominantly microcalcifications), cystic degeneration, and blood flow distribution of peripheral or mixed blood flow signals. In our patient, the left neck mass was not well characterized on the ultrasound image, showing uniform and consistent iso- or slightly hypo-thyroid-like echoes with abundant blood flow signals and isolated oval echoes on the ultrasound. We consider ectopic thyroid to be more likely.

Second, painless masses highly suspected by ultrasound to be ectopic thyroid should be combined with nuclear (99mtco4-, 123I, or 131I) planar imaging to improve diagnostic efficacy. For cystic-solid and solid lesions with abundant blood flow signals, FNA is necessary for further diagnosis when malignancy cannot be excluded. In addition, CT and MRI have value in helping to determine the extent and location of ectopic tissue and increasing the suspicion of malignancy in cases of cervical lymph node enlargement (2).

### 3.3 Pathologic features and molecular level

Histopathologic examination remains the current gold standard for confirming the nature of a neck mass. In addition, fine needle aspiration cytology (FNAC), which is the only way to differentiate between benign and malignant lesions preoperatively (15), helps to diagnose ectopic tissue to some extent, but may sometimes be misdiagnostic or nondiagnostic, especially in cases presenting as cystic masses.

There have also been advances in recent years regarding the study of ectopic thyroid cancer with lymph node metastases at the molecular level, but further validation is still needed. Lin et al. demonstrated that immunohistochemistry (IHC) of the mutant BRAFV600E protein (mBRAF) could be used to identify metastatic follicular carcinomas derived from thyroid follicular inclusion bodies (TFIs) containing mBRAF in PTCs by comparing the results of PCR analysis of the BRAFV600E gene (16); Chieko Otsubo et al. demonstrated that immunofluorescence (IF) analysis of 53BP1 expression in cases other than BRAFV600E mutant PTC may be an excellent adjunctive technique for distinguishing whether a well-formed TFI in the cervical lymph nodes is metastatic carcinoma or ectopic thyroid tissue (ETT) by comparing the mutation analysis of primary thyroid cancer (7). In addition, it has been found that cranial ectopic thyroid cancer, *in situ* thyroid cancer and metastatic thyroid cancer do not have the same molecular expression (17): AKT and mTOR are highly expressed in cranial ectopic thyroid cancer, whereas MMP-9 is highly expressed in metastatic thyroid cancer.

Notably, case reports predominate in the description of characteristic manifestations of ectopic thyroid cancer. Taking into account the literature and the available clinical data, the author believes that the following features are helpful in making a final differential diagnosis of our case:(1) A painless mass is palpated in the lateral neck on physical examination;(2) Ultrasound demonstrated isolated oval isoechoic thyroid-like echoes rather than the more typical signs of suspicious lymph nodes;(3) In terms of gross morphology, the lateral neck mass had an intraoperative gross appearance similar to that of the thyroid gland, with a more vascularized surface but no connection to the thyroid gland;(4) Encapsulated papillary carcinoma was seen on histopathologic examination, with no lymphoid tissue seen within the envelope and only one metastatic lymph node seen at its margin. This pathologic feature can be considered as one of the three morphologic manifestations of “lateral cervical ectopic thyroid” – ectopic tissue present in the cervical lymph nodes or their remnants. At this time, due to the high degree of differentiation of most papillary thyroid carcinomas, the morphology of their lymph node metastases can have no obvious heterogeneity under the microscope, and show similar pathologic features to normal thyroid tissue. Similarly, Batsakis also believes that any “lateral neck ectopic thyroid”, however normal it may appear, is a metastasis of the primary thyroid cancer rather than a developmental abnormality (3). The absence of lymph node tissue in this case may be the complete replacement of lymphatic tissue by well-differentiated thyroid carcinoma (4). In conclusion, we consider this patient’s lateral neck mass as metastatic lateral neck ectopic thyroid carcinoma.

### 3.4 Treatment and prognosis

Lateral neck ectopic thyroid cancer is mainly treated by surgery at home and abroad, but there is no evidence-based guideline for the optimal treatment standard, and individualized treatment should be performed. Notably, Hongjian Yang found primary thyroid cancer in the postoperative pathology of 59 patients with “lateral cervical ectopic thyroid” who had *in situ* thyroidectomy (3). Fifty-three of these cases belonged to thyroid tissue found within the lateral lymph nodes of the carotid sheath, and six cases were thyroid tissue with no lymph node structures seen in the lateral carotid sheath. This suggests that the operator needs to consider the potential for malignant transformation and take a more aggressive surgical approach to treatment if necessary:

For patients with strong preoperative suspicion of ectopic thyroid cancer, (1) when their *in situ* thyroid gland did not show obvious signs of malignancy preoperatively, a staged approach could be adopted (8), that is, the lateral neck mass resection is performed first, and after the ectopic tissue is confirmed by excision biopsy as malignant lesions, at least complete ipsilateral thyroidectomy + central group lymph node dissection + ipsilateral neck lymph node dissection is performed, so as to provide both safe and effective tumor treatment.; (2) And for patients whose preoperative period has suggested malignant lesions of the thyroid gland *in situ*, total bilateral thyroidectomy + central group lymph node dissection + ipsilateral cervical lymph node dissection is recommended, and the need for postoperative radiotherapy is decided.

If the definite lateral neck mass is a lymph node metastatic cancer, radioactive iodine nail-clearing therapy is recommended for patients with DTC whose risk of recurrence is stratified as intermediate to high according to evidence-based guidelines (18). Meanwhile, all postoperative follow-ups require attention to their TG levels to monitor recurrence.

In conclusion, it is not possible to accurately differentiate primary ectopic thyroid carcinoma or lymph node metastatic carcinoma from pathologic masses in the lateral neck by relying solely on clinical features and imaging, and preoperative cytology can help diagnose the benignness or malignancy of the lesions to a certain extent, The current gold standard for diagnosis remains histopathologic examination. It is noteworthy that some research progress has been made in recent years at the molecular level for the differentiation of the two, but further research and validation are still needed. Lymph node metastatic carcinoma is more common among thyroid malignant tumors, and its treatment has reached consensus, And there are no standard evidence-based guidelines for the optimal treatment of lateral neck ectopic thyroid cancer. Treatment should be individualized accordingly, taking into account the possibility of malignant transformation and aggressive treatment if necessary, accordingly, more attention should be paid to the preoperative vocal cord movement and the protection of recurrent laryngeal nerve during operation (19).

## Data Availability

The datasets presented in this article are not readily available because of ethical and privacy restrictions. Requests to access the datasets should be directed to the corresponding author.
